# Germline inactivation of tumor suppressor *BAP1* is associated with white spotting

**DOI:** 10.1172/JCI195809

**Published:** 2026-01-02

**Authors:** Ching-Ni Njauw, Zhenyu Ji, David I. Latoni, Jose Mari Villa-Gonzalez, Shelley McCormick, Raj Kumar, Dmitrii Usoltsev, Mykyta Artomov, Boyi Gan, Hensin Tsao

**Affiliations:** 1 Wellman Center for Photomedicine, Massachusetts General Hospital (MGH), Boston, Massachusetts, USA.; 2 Department Dermatology, Univ Puerto Rico School of Medicine, San Juan, Puerto Rico.; 3 Department Dermatology, Cruces University Hospital, Bizkaia, Spain.; 4 Mass General Brigham Cancer Institute, Boston, Massachusetts, USA.; 5 Division Hematology/Oncology, Department Medicine, MGH, Boston, Massachusetts, USA.; 6 The Steve and Cindy Rasmussen Institute for Genomic Medicine, and Dept Pediatrics, Nationwide Children’s Hospital, Columbus, Ohio, USA.; 7 Department Experimental Radiation Oncology, MD Anderson Cancer Center, Houston, Texas, USA.

**Keywords:** Dermatology, Genetics, Genetic diseases, Melanoma, Skin

## Abstract

Inherited BAP1 mutations cause melanoma and other cancers and can also lead to white hair patches or skin spots due to pigment cell loss.

**To the Editor:** The BAP1 tumor predisposition syndrome (BAP1-TPDS), caused by germline variants in *BAP1*, increases susceptibility to multiple malignancies, including cutaneous melanoma (CM) and uveal melanoma (1). While *BAP1* functions as a putative melanoma tumor suppressor, its full role in melanocyte survival and proliferation is poorly understood. We describe a white spotting phenotype in patients with germline *BAP1* variants. Case 1 is a female patient with a germline p.Asp236Glyfs*7 variant who developed CM and breast cancer at ages 49 and 52, respectively (Figure 1A). She had a white forelock since birth but lacked eye or hearing problems. Direct visual and Wood’s light inspection of 13 more *BAP1* variant carriers identified another 7 patients with chronic poliosis or depigmentation (Figure 1A).

To establish a biological link, we examined transcriptomes from normal human melanocytes (NHM; *n =* 308 samples from 35 patients) and tumors in the TCGA_SKCM (*n =* 363 samples) and the Lund melanoma cohorts (*n =* 214 samples) to determine if correlations between BAP1 expression levels and melanocyte lineage specifying genes exist (Supplemental Table 1 and Supplemental Figure 1A; supplemental material available online with this article; https://doi.org/10.1172/JCI195809DS1). Among the piebaldism and Waardenburg syndrome genes (Supplemental Figure 1B), *SOX10* and *BAP1* expression exhibited the highest correlation in all 3 datasets, while *KIT* and *MITF* levels also correlated with *BAP1* levels, albeit weaker (Supplemental Figure 1B).

We then ranked all genes by their *BAP1* correlation coefficient and performed Gene Ontology (GO) analysis (Figure 1B). The GO biological process terms most positively correlated with *BAP1* expression were PIGMENTATION (Figure 1B and Supplemental Table 1) and RESPONSE TO IL-4 for NHM, PIGMENT METABOLIC PROCESS and PIGMENTATION for the TCGA_SKCM samples and PIGMENTATION and ENDOSOMAL TRANSPORT for the Lund specimens. PIGMENT GRANULE ranked among the top 2 GO-cellular compartment terms in all 3 datasets. Additionally, pigmentation disorders also ranked at the top of the Human Phenotype Ontology database (Figure 1B) among *BAP1*-correlated genes for NHM and melanoma tumors. HOMER analysis of the top 500 genes most correlated with *BAP1* (Figure 1C) showed motif enrichments for MITF and TFE3, transcription factors regulating melanocyte lineage specification (2), in all 3 cohorts. *BAP1* levels were also highly correlated with a curated set of 110 known or putative MITF targets (Supplemental Figure 1C and Supplemental Figure 2).

Functionally, we previously showed (3) that *BAP1* depletion reduced melanoma cell viability and tumorigenicity, paradoxical to its putative suppressor role. With DepMap, we confirm that CRISPR-mediated deletion of *BAP1* in 96 melanoma lines is associated with a loss of melanoma viability (Supplemental Figure 3). We subsequently depleted *BAP1* in 3 melanoma cell lines and observed marked reductions in the protein levels of Sox10 and Mitf, cellular growth, and cellular pigmentation (Figure 1D and Supplemental Figure 4). Using *Tyr:CreA* with *Bap1*^fl/fl^ mice, we also tested if melanocyte-specific *BAP1* deletion can lead to a white spotting phenotype in vivo. No notable skin phenotypes were identified in either parent strains or the *Tyr:CreA*/*Bap1*^fl/+^ mice. However, all *Tyr:CreA*/*Bap1*^fl/fl^ had depigmented paws (Figure 1E; red circle), and 3 of 4 had a white abdominal spot (Figure 1E). Compared with *Bap1*^fl/fl^ mice, *Tyr:CreA*/*Bap1*^fl/fl^ animals exhibited significant follicular melanocyte dropout in the depigmented zone (Figure 1F), ear, and dorsal foot; no tumors developed in any of the mice.

Nontumorigenic phenotypes have not been well characterized in the BAP1-TPDS. We provide a patient-to-mouse description of a nontumor phenotype for the BAP1-TPDS, and our evidence suggests that *BAP1* may impinge on the migration, differentiation, survival, and melanization of follicular melanocytes. While the case series is small, the extreme rarity of the BAP1-TPDS precludes large-scale analysis, such as genotype-phenotype correlations. A pigmentation phenotype has not been described in previous murine models of *Bap1* inactivation, as *Bap1^–/–^* embryos do not survive past day E9.5 (4). Heterozygous *Bap1*^+/–^ are more susceptible to myeloid transformation (4) and mesotheliomas (5). Suppression of *Bap1* in *Xenopus* during development also disrupts cell identity and ectodermal, mesodermal, and neural crest abnormalities (6). Thus, *BAP1* may regulate melanocyte migration, identity, and pigmentation. Lastly, in tuberous sclerosis, hypopigmented macules and tumors occur with tuberin loss and mTOR activation, phenotypes reversed with topical rapamycin (7), thereby raising the possibility for rapamycin in *BAP1*-associated neoplasia.

## Funding support

- Donors to the Innovations in Melanoma Care Fund.
- The Millennium Fund for Melanoma.
- The Richard Allen Johnson, MD Endowed Chair in Dermatology.

## Supplementary Material

Supplemental data

Unedited blot and gel images

Supplemental table 1

Supporting data values

## Figures and Tables

**Figure 1:**
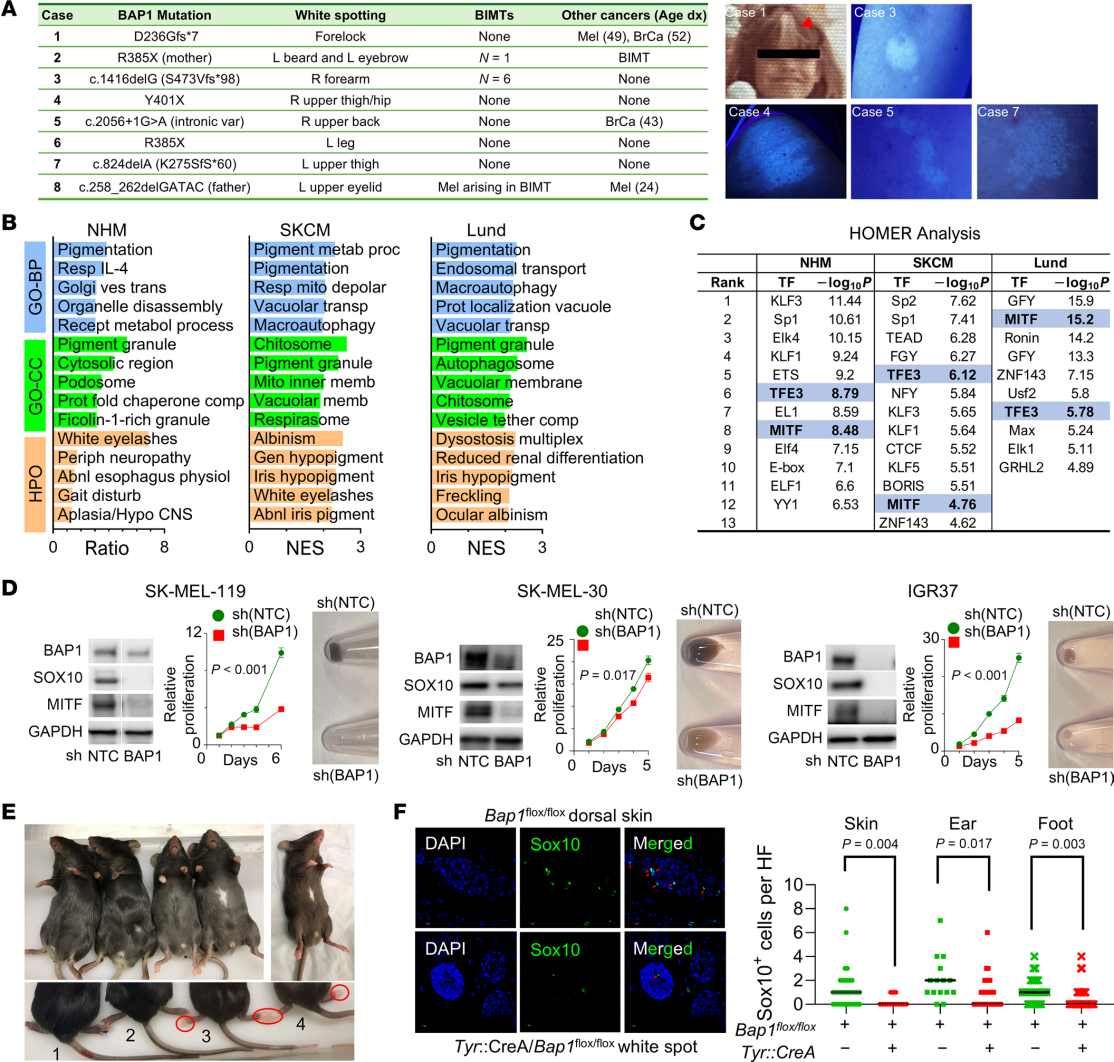
*BAP1* inactivation and pigmentation. (**A**) Patient details and images of white spotting (demonstrated by Wood’s light in Cases 3,4,5,7). (**B**) Top genes correlated with *BAP1* expression in normal human melanocytes (NHMs), the TCGA_SCKM and Lund cohorts, as ranked by Spearman coefficient, were subjected to Gene Ontology Biological Processes (GO-BP), GO-Cellular Compartment (GO-CC), and Human Phenotype Ontology (HPO) analysis; all GO and HPO analyses exhibited FDR < 0.05. (**C**) The 500 genes most correlated with *BAP1* expression were subjected to HOMER analysis. Pigmentation-related transcription factors, MITF and TFE3, are highlighted in blue. (**D**) Suppression of Bap1 by sh(BAP1) in 3 melanoma cell lines leads to decreases in SOX10 and MITF, reduced cellular proliferation (Day 0 = 1; *P* values determined by *t* test on Day 5), and pigmentation loss. (**E**) Skin phenotypes of various *Bap1* genotypes. Animal 1 (*Bap1*^fl/fl^) and Animal 2 (*Tyr*:*CreA*/*Bap1*^fl/WT^) have no visible phenotype. Animals 3–5 (*Tyr*:*CreA*/*Bap1*^fl/fl^) showed depigmentation of the fore- and hindpaws (red circles) while animals 4 and 5 had white spotting on the midabdomen. (**F**) Melanocyte quantitation (Sox10+ cells per hair follicle (HF)) shows significant reductions in the number of Sox10+ melanocytes (red arrows) in the depigmented abdominal area, ear, and dorsal paw of *Tyr*:CreA/*Bap1*^fl/fl^ mouse compared with the *Bap1*^fl/fl^ mouse (2-tailed *t* test).

## Version 1 01/02/2026

Electronic publication
