# Supplementary material for: Germline inactivation of tumor suppressor BAP1 is associated with white spotting
Source: J Clin Invest. 2026 Jan 2;136(1):e195809. doi: 10.1172/JCI195809 (PMC12721880; doi:10.1172/JCI195809)
Supplement: Supplemental data [file jci-136-195809-s336.pdf]

A.

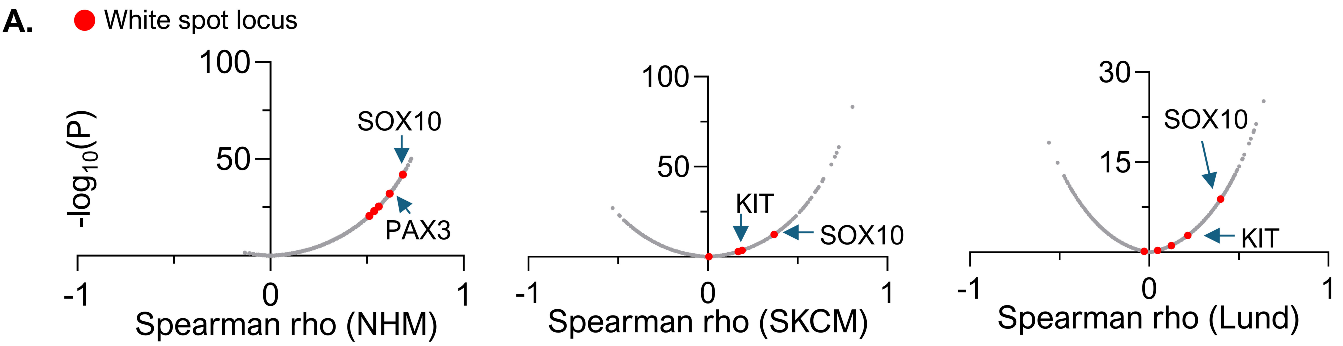

B.

		NHM		SKCM		Lund	
**Condition**	**Gene**	rho	**p**	rho	**p**	rho	**p**
Piebaldism	*KIT*	0.512	**2.67E-21**	0.190	**2.63E-04**	0.215	**1.52E-03**
WS 1	*PAX3*	0.618	**9.70E-33**	0.170	**1.15E-03**	-0.028	0.68
WS 2A	*MITF*	0.561	**4.44E-26**	0.166	**1.47E-03**	0.214	0.07
WS 2E	*SOX10*	0.685	**1.35E-42**	0.370	**3.41E-13**	0.399	**1.39E-09**
WS 3	*PAX3*	0.618	**9.70E-33**	0.170	**1.15E-03**	-0.028	0.68
WS 4A	*EDNRB*	0.538	**8.74E-24**	0.0055	0.92	0.122	0.07
WS 4B	*EDN3*	NA	NA	-0.085	0.11	0.047	0.49

C.

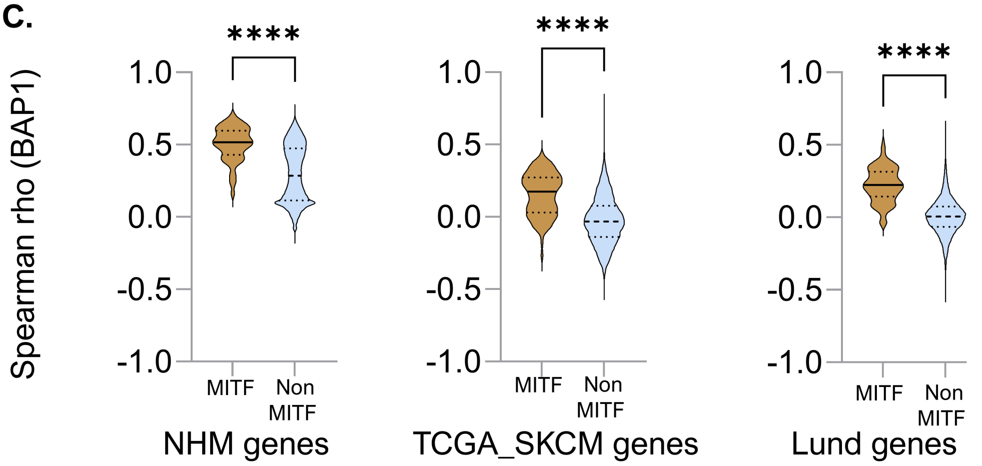

Figure S1. (A) Volcano plot correlating expression levels of *BAP1* with all genes in the NHM, TCGA_SKCM and Lund datasets. **(B)** Specific Spearman correlations between BAP1 and genes known to cause white spotting phenotypes in humans. **(C)** A curated set of 110 MITF targeted genes (Pigment Cell Melanoma Res. 2008 Dec;21(6):665-76) also exhibited, on average, higher Spearman correlations compared to all other non-MITF target genes in all three datasets; Abbr: WS, Waardenburg; NHM, normal human melanocytes; **** p<0.0001 by unpaired T test

Rank	Motif	Name	P-value	log P-pvalue	q-value (Benjamini)
1	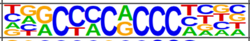	KLF3(Zf)/MEF-Klf3-ChIP-Seq(GSE44748)/Homer	1e-4	-1.144e+01	0.0047
2	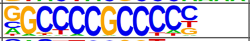	Sp1(Zf)/Promoter/Homer	1e-4	-1.061e+01	0.0054
3	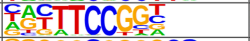	Elk4(ETS)/Hela-Elk4-ChIP-Seq(GSE31477)/Homer	1e-4	-1.015e+01	0.0057
4	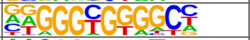	KLF1(Zf)/HUDEP2-KLF1-CutnRun(GSE136251)/Homer	1e-4	-9.244e+00	0.0106
5	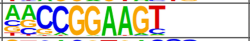	ETS(ETS)/Promoter/Homer	1e-3	-9.196e+00	0.0106
6	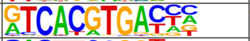	TFE3(bHLH)/MEF-TFE3-ChIP-Seq(GSE75757)/Homer	1e-3	-8.788e+00	0.0112
7	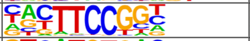	Elk1(ETS)/Hela-Elk1-ChIP-Seq(GSE31477)/Homer	1e-3	-8.587e+00	0.0117
8	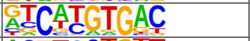	MITF(bHLH)/MastCells-MITF-ChIP-Seq(GSE48085)/Homer	1e-3	-8.476e+00	0.0117
9	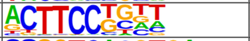	Elf4(ETS)/BMDM-Elf4-ChIP-Seq(GSE88699)/Homer	1e-3	-7.147e+00	0.0385
10	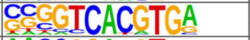	E-box(bHLH)/Promoter/Homer	1e-3	-7.102e+00	0.0385
11	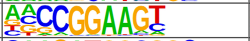	ELF1(ETS)/Jurkat-ELF1-ChIP-Seq(SRA014231)/Homer	1e-2	-6.600e+00	0.0544
12	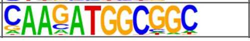	YY1(Zf)/Promoter/Homer	1e-2	-6.531e+00	0.0544

NHM

Rank	Motif	Name	P-value	log P-pvalue	q-value (Benjamini)
1	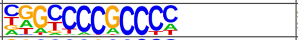	Sp2(Zf)/HEK293-Sp2.eGFP-ChIP-Seq(Encode)/Homer	1e-3	-7.621e+00	0.2157
2	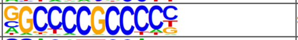	Sp1(Zf)/Promoter/Homer	1e-3	-7.410e+00	0.2157
3	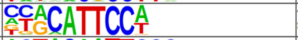	TEAD1(TEAD)/HepG2-TEAD1-ChIP-Seq(Encode)/Homer	1e-2	-6.281e+00	0.2745
4	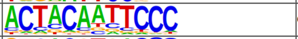	GFY(?)/Promoter/Homer	1e-2	-6.270e+00	0.2745
5	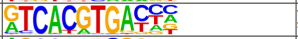	TFE3(bHLH)/MEF-TFE3-ChIP-Seq(GSE75757)/Homer	1e-2	-6.120e+00	0.2745
6	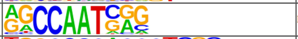	NFY(CCAAT)/Promoter/Homer	1e-2	-5.836e+00	0.2745
7	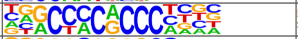	KLF3(Zf)/MEF-Klf3-ChIP-Seq(GSE44748)/Homer	1e-2	-5.652e+00	0.2745
8	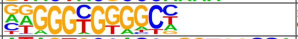	KLF1(Zf)/HUDEP2-KLF1-CutnRun(GSE136251)/Homer	1e-2	-5.642e+00	0.2745
9	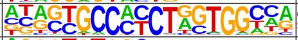	CTCF(Zf)/CD4+-CTCF-ChIP-Seq(Barski_et_al.)/Homer	1e-2	-5.517e+00	0.2745
10	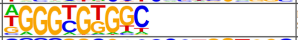	KLF5(Zf)/LoVo-KLF5-ChIP-Seq(GSE49402)/Homer	1e-2	-5.507e+00	0.2745
11	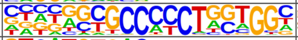	BORIS(Zf)/K562-CTCFL-ChIP-Seq(GSE32465)/Homer	1e-2	-5.507e+00	0.2745
12	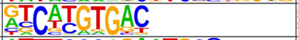	MITF(bHLH)/MastCells-MITF-ChIP-Seq(GSE48085)/Homer	1e-2	-4.760e+00	0.3142
13	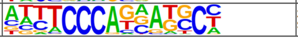	ZNF143|STAF(Zf)/CUTLL-ZNF143-ChIP-Seq(GSE29600)/Homer	1e-2	-4.617e+00	0.3345

TCGA_SKCM

Rank	Motif	Name	P-value	log P-pvalue	q-value (Benjamini)
1	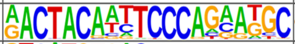	GFY-Staf(?,Zf)/Promoter/Homer	1e-6	-1.587e+01	0.0001
2	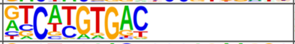	MITF(bHLH)/MastCells-MITF-ChIP-Seq(GSE48085)/Homer	1e-6	-1.524e+01	0.0001
3	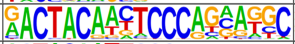	Ronin(THAP)/ES-Thap11-ChIP-Seq(GSE51522)/Homer	1e-6	-1.424e+01	0.0001
4	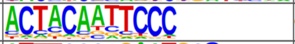	GFY(?)/Promoter/Homer	1e-5	-1.328e+01	0.0002
5	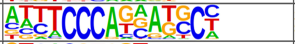	ZNF143|STAF(Zf)/CUTLL-ZNF143-ChIP-Seq(GSE29600)/Homer	1e-3	-7.145e+00	0.0694
6	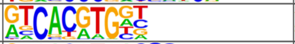	Usf2(bHLH)/C2C12-Usf2-ChIP-Seq(GSE36030)/Homer	1e-2	-5.800e+00	0.2219
7	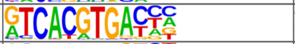	TFE3(bHLH)/MEF-TFE3-ChIP-Seq(GSE75757)/Homer	1e-2	-5.782e+00	0.2219
8	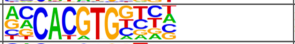	Max(bHLH)/K562-Max-ChIP-Seq(GSE31477)/Homer	1e-2	-5.244e+00	0.2902
9	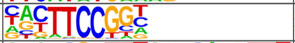	Elk1(ETS)/Hela-Elk1-ChIP-Seq(GSE31477)/Homer	1e-2	-5.106e+00	0.2962
10	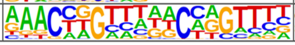	GRHL2(CP2)/HBE-GRHL2-ChIP-Seq(GSE46194)/Homer	1e-2	-4.893e+00	0.3298

Lund

Figure S2. HOMER analysis of top 500 genes whose expression was most positively correlated with BAP1 expression by Spearman rho. Abbrev: NHM, normal human melanocyte

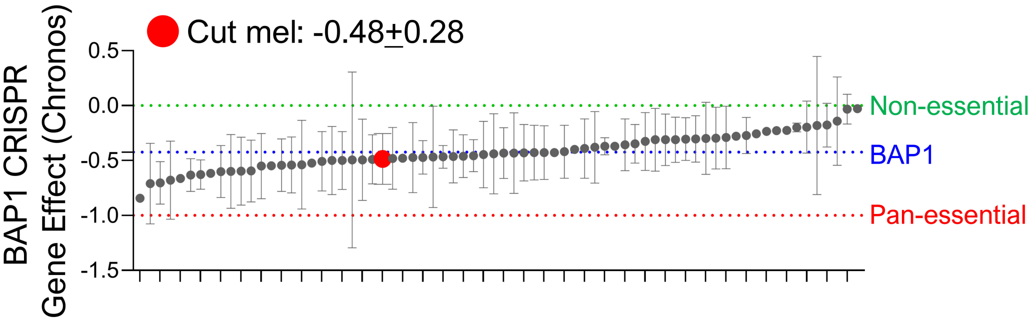

Figure S3. Effect of BAP1 deletion in the DepMap database among all cell lines. The average gene effect for melanoma lines is indicated by the red dot. The average effect across the lines is shown the the blue BAP1 line. A score of “0” means that CRISPR-mediated BAP1 deletion had no effect on viability while a score of “-1” suggests that the gene is pan essential for viability.

ONLINE SUPPLEMENTARY METHODS

Germline *BAP1* inactivation is associated with white spotting

Ching-Ni Njauw[1]* (0000-0003-4543-3769), Zhenyu Ji[1]* (0000-0002-3353-6376), David I. Latoni[2]* (0000-0001-6104-7601), Jose Mari Villa Gonzalez[3], Shelley McCormick[4], Raj Kumar[5], Dmitrii Usoltsev[6], Mykyta Artomov[6], Boyi Gan[7], Hensin Tsao[1,4] (0000-0002-2204-2071)

[1] Wellman Center for Photomedicine, MGH, Boston, MA
[2] Dept Dermatology, University of Puerto Rico School of Medicine, San Juan, Puerto Rico
[3] Dept Dermatology, Cruces University Hospital, Spain
[4] Mass General Brigham Cancer Center, Boston, MA
[5] Div Hematology & Oncology, Dept Medicine, MGH, Boston, MA
[6] The Steve and Cindy Rasmussen Institute for Genomic Medicine, Nationwide Children's Hospital, Columbus, OH, 43205. Dept Pediatrics, Ohio State University College of Medicine, Columbus, OH, 43205
[7] Dept Exp Rad Oncology, The University of Texas MD Anderson Cancer Center, Houston, TX
*These authors contributed equally based on extent to scientific input and writing.

Corresponding author:
Dr. Hensin Tsao
htsao@mgh.harvard.edu
Massachusetts General Hospital
Edwards 211
50 Blossom Street
Boston, MA 02114

The authors declare no competing interests

MATERIALS AND METHODS:

Western blot
Cells were lysed with RIPA buffer supplemented with protease and phosphatase inhibitor cocktails (Selleck). Lysed cells were left on ice for 10 min and then centrifuged at 10,000 rpm at 4° ˚C for 10 min. The supernatant was collected and stored at -80ºC. An equal amount of the total protein (5-20 µg) was loaded on 4-20% polyacrylamide gels (Bio-Rad, Hercules, CA) and run in Tris-Glycine running buffer. Gels were transferred to polyvinylidene difluoride membranes using a trans-blot turbo transfer system (Bio-Rad, Hercules, CA). The blots were blocked in 5% milk in TBS-Tween for 1 hour and then incubated with the primary antibody overnight. Secondary antibody conjugated with horseradish peroxidase was incubated for 1 hr at room temperature. Membranes were then incubated with a 1:1 mixture of the enhanced chemiluminescence solution (Bio-Rad, Hercules, CA) for 5 min, protected from light. Images were obtained using the ChemiDoc MP Image system (Bio-Rad, Hercules, CA). The signal intensity was quantified using ImageJ analysis.

Cell lines
The SK-MEL-119, SK-MEL-30, and IGR37 cell lines are lab reagents with details in the public domain (descriptions in Cellosauras: https://www.cellosaurus.org).

Antibodies:
BAP1(C-4): from Santa Cruz, mouse, cat# sc-28383. And from Abcam, rabbit, cat# ab199396.
SOX10(A-2): from Santa Cruz, rabbit, cat# sc-365692
MITF (C-5): from David Fisher's lab, mouse.
SOX10 immunostaining: Sox10 (D5V9L): from Cell Signaling, rabbit, cat# 89356.

Immunofluorescence staining
Skin was collected, fixed with formalin, and embedded. 5 µm sections were cut and baked for staining. Tissue sections were deparaffinized and rehydrated. Permeabilization was achieved using 0.2% Triton X-100 in PBS, followed by antigen retrieval according to the manufacturer's protocol for the specific primary antibody. Tissue sections were blocked by 10% goat serum with 5% BSA in PBS for 1 hr at room temperature. Following blocking, sections were incubated overnight at 4°C with an anti-Sox10 primary antibody (Cell Signaling, Danvers, MA). The next day, the excess primary antibody was removed by thorough washing, and sections were incubated with an Alexa Fluor 488-conjugated goat anti-rabbit secondary antibody (Thermo Fisher Scientific, Waltham, MA) for 1 hr at room temperature. After washing to remove unbound secondary antibody, tissue sections were mounted with DAPI-containing mountant and allowed to cure for 24 hours. Fluorescent images were captured using an FV-1000 confocal microscope (Olympus, Tokyo, Japan).

Animals
All animal procedures were approved by the Hospital Institutional Animal Care and Use Committee (IACUC) and were conducted per the guidelines outlined by the National Research Council's Guide for the Care and Use of Laboratory Animals. *Bap1*$^{flox/flox}$ mice were obtained from Dr. Gan[1]. Female *Tyr::CreA* mice were obtained from Jax Labs (strain 029788) and were crossed with male *Bap1*$^{flox/flox}$ or with *Bap1*$^{flox/+}$ to generate *Tyr::CreA*, *Bap1*$^{flox/flox}$, or *Tyr::CreA*/*Bap1*$^{flox/+}$ mice. Sex could not be treated fully as an independent variable since the *Tyr::CreA* transgene is integrated on the X chromosome; in other words, there is no heterozygous state for the *Tyr::Cre* in male mice. To minimize culling, sex was used as a selectable marker, and only male offspring were collected for further study. Given that the human phenotype occurs in both sexes and the BAP1 gene is located on an autosomal chromosome, the BAP1 effect is unlikely to be determined.

Transfection and transduction
293T cells were transiently transfected with shRNAs against human BAP1 (Sigma) with MISSION® Lentiviral Packaging Mix (Sigma, cat. #SHP001) using Lipofectamine 2000 transfection reagent (Thermo Fisher Scientific, cat. #11668027) according to the manufacturer's protocol. To perform transfections, a mixture of 0.5 µg shRNA and 5 µL packaging mix was first made in a total volume of 250 µL at room temperature in Opti-MEM (Life Technologies, cat. #31985088). Lipofectamine 2000 reagent 3 µLwas diluted in a separate volume of 250 µL room temperature Opti-MEM, transferred to the tube containing the shRNA/packaging mix, then incubated for 15 minutes at room temperature. After the incubation, the combined mixture was added to a well of a 6-well plate, and 5×10^4 293T cells in 500 µL Opti-MEM media were then added. Transfected cells were incubated at 37°C for 48 hours before collecting viral supernatant. The medium supernatant with secreted viral particles was harvested and filtered through a 0.45 µm filter(Fisher Scientific, cat. #09-720-4) and then added to melanoma cells along with Polybrene (8 mg/mL; Fisher Scientific, cat. #NC9840454) and left for 20 hours. Two days post-transduction, the medium was replaced with a complete puromycin medium (1 mg/mL; Enzo, cat. #BML-GR312-0250) for positive selection. BAP1 knockdown was confirmed by Western blot.

Growth curves
Growth measurements across multiple time points (Days 0–5) and three independent cell lines (SK-MEL-119, SK-MEL-30, and IGR37) were measured daily using CellTiter-Glo® in triplicate wells (1000 cells per well). Cell proliferation data were analyzed using a linear mixed effects model (LMM) to assess differences in growth trajectories between experimental groups (e.g., sh^{BAP1} *vs* sh^{NTC}). The data were first reshaped to a long format, and missing values were linearly interpolated where necessary. Each replicate was treated as a random effect to account for repeated measurements, while time (Days), group, and their interaction were modeled as fixed effects. LMM was chosen over traditional repeated-measures ANOVA due to its robustness to missing data, flexibility in modeling unbalanced designs, and ability to account for inter-sample variability. A significant interaction between time and group indicated divergent growth trajectories between conditions. All analyses were performed using the "statsmodels" package in Python.

Study Approval
De-identified patient information for the BAP1 cases was covered under Mass General Brigham (MGB) protocol 2022P002105. Written informed consents were received for the use of the photographs, and the records of informed consent have been retained. The mice breeding studies were covered under the MGH IACUC protocol 2013N000110.

Statistical analysis.
All statistical analyses were two-tailed, and a p-value of <0.05 was considered statistically significant. Continuous variables were analyzed using parametric tests (Student's t-test or one-way ANOVA) when data were normally distributed, and non-parametric tests (Mann–Whitney U test or Kruskal–Wallis test) when normality assumptions were not met. Categorical variables were compared using parametric tests (Pearson's χ^2 test) or non-parametric alternatives (Fisher's exact test) as appropriate. Correlation between continuous variables was assessed using Pearson's correlation coefficient (r) for normally distributed data or Spearman's rank correlation coefficient (ρ) for non-normally distributed data. Statistical analyses were performed using standard statistical software, and all tests were two-tailed with significance set at $p < 0.05$.

Databases and analysis
Normal human melanocytes (NHM). mRNA expression data from 308 human skin melanocyte samples (35 patients) in cBioPortal were subjected to *BAP1* correlation using the CO-EXPRESSION module.

SKCM_TCGA. mRNA expression from the TCGA_SKCM in cBioPortal ("Skin Cutaneous Melanoma (TCGA, PanCancer Atlas)") with "Complete Samples (363)" was subjected to correlation analysis using the CO-EXPRESSION module.

Lund. mRNA expression data from the Lund cohort were obtained from the Gene Expression Omnibus database, accession GSE65904 (https://www.ncbi.nlm.nih.gov/geo/query/acc.cgi?acc=GSE65904), comprising N=214 samples. The expression values were subjected to quantile normalization using the normalizeBetweenArrays function in the R[2] package limma (version 3.54.2)[3]. All correlation analyses were conducted in R.

Gene ontology and Human Phenotype Ontology analyses were performed using WEB-based GEne SeT AnaLysis Toolkit (WebGestalt v.2024; https://www.webgestalt.org/). For the TCGA_SKCM and Lund datasets, the sets of genes and Spearman rankings (Supplementary Table) were subjected to Gene Set Enrichment Analysis. However, for the normal human melanocytes, there was a right skew that rendered most correlations to be positive, thereby also skewing the GSEA. Thus, for the NHM dataset, we used over-representation analysis of the top 1000 most correlated genes.

HOMER. The motif discovery tool HOMER was used to identify potential transcriptional factors responsible for *BAP1*-related gene expression. The HOMER(V5.1) software was downloaded from its official website (https://homer.ucsd.edu/), and was run locally. The top 500 most positively correlated genes were used as input. HOMER's findMotifs.pl script was implemented with motif lengths of 8 and 10 base pairs, and a search space of 400 bp upstream and 100 bp downstream of the transcription start site. Known motifs from the HOMER database were manually scanned to identify matches to previously characterized transcription factor binding sites. The identified transcription factors were ranked based on their p-values.

Data availability
All data and software used in this study are in the public domain as outlined above in the "Databases and analysis" section. Original data for the figures are shown in the "195809-JCI-RL-DN-2 Data for Figures" Excel file.

1. Dai, F. et al. BAP1 inhibits the ER stress gene regulatory network and modulates metabolic stress response. Proc Natl Acad Sci U S A 114, 3192-3197, doi:10.1073/pnas.1619588114 (2017).

2. Team, R. C. R: A language and environment for statistical computing. R Foundation for Statistical Computing, Vienna, Austria, <https://www.R-project.org/> (2021).

3. Ritchie, M. E. et al. limma powers differential expression analyses for RNA-sequencing and microarray studies. Nucleic acids research 43, e47, doi:10.1093/nar/gkv007 (2015).

4. John M Elizarraras, Yuxing Liao, Zhiao Shi, Qian Zhu, Alexander R Pico, Bing Zhang, WebGestalt 2024: faster gene set analysis and new support for metabolomics and multi-omics, Nucleic Acids Research, 2024, gkae456

5. Liao, Y., Wang, J., Jaehnig, E., Shi, Z., Zhang, B. WebGestalt 2019: gene set analysis toolkit with revamped UIs and APIs, Nucleic Acids Research, gkz401

6. Wang, J., Vasaikar, S., Shi, Z., Greer, M., & Zhang, B. WebGestalt 2017: a more comprehensive, powerful, flexible and interactive gene set enrichment analysis toolkit. Nucleic Acids Research.

7. Wang, J., Duncan, D., Shi, Z., Zhang, B. (2013). WEB-based GEne SeT AnaLysis Toolkit (WebGestalt): update 2013. Nucleic Acids Res, 41 (Web Server issue), W77-83.

8. Zhang, B., Kirov, S.A., Snoddy, J.R. (2005). WebGestalt: an integrated system for exploring gene sets in various biological contexts. Nucleic Acids Res, 33(Web Server issue), W741-748.
