# Supplementary material for: Germline inactivation of tumor suppressor BAP1 is associated with white spotting
Source: J Clin Invest. 2026 Jan 2;136(1):e195809. doi: 10.1172/JCI195809 (PMC12721880; doi:10.1172/JCI195809)

SK-MEL-119

Gel 1 long exposure

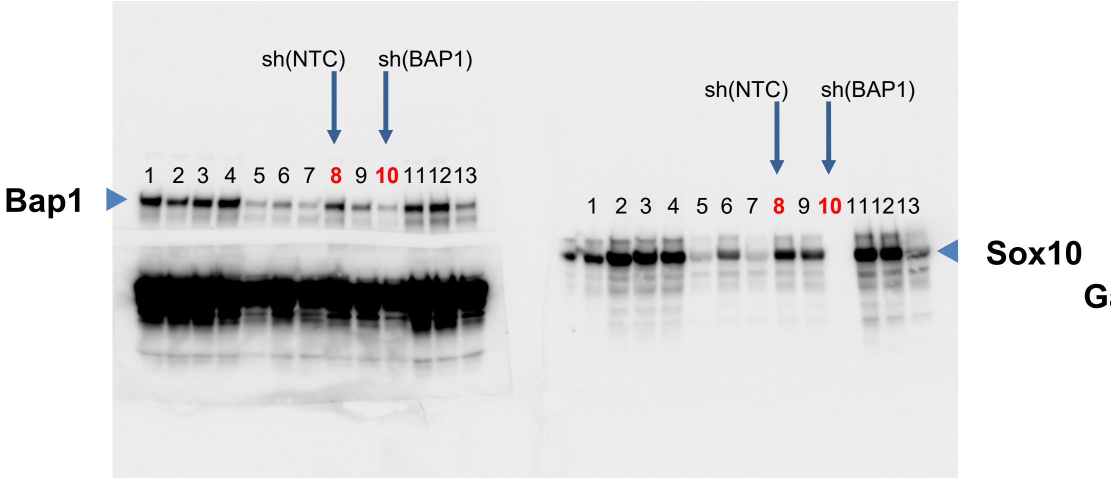

Gel 1 short exposure

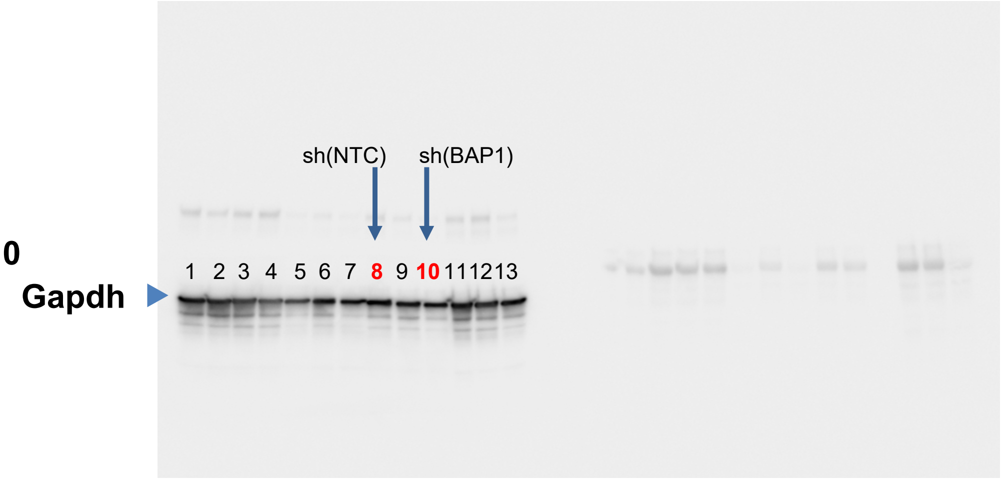

Gel 2

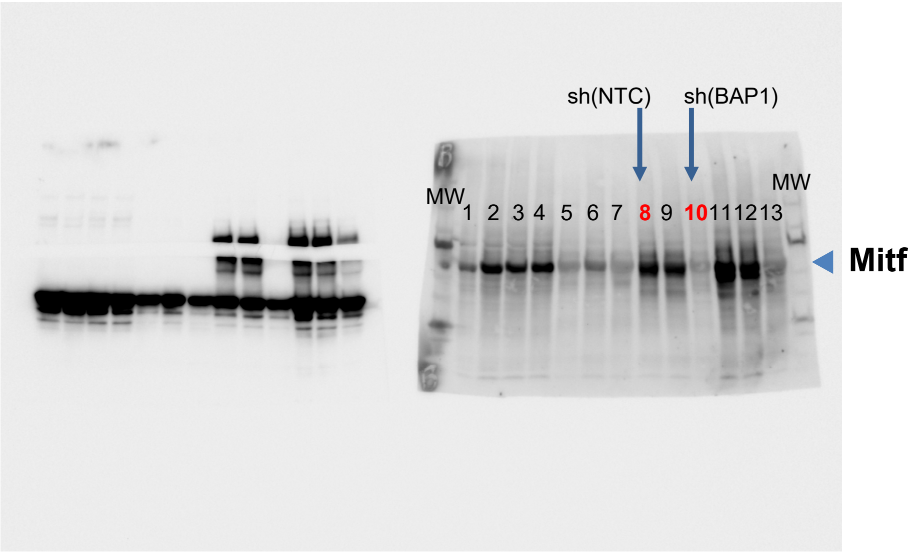

Fig 1D. SK-MEL-119

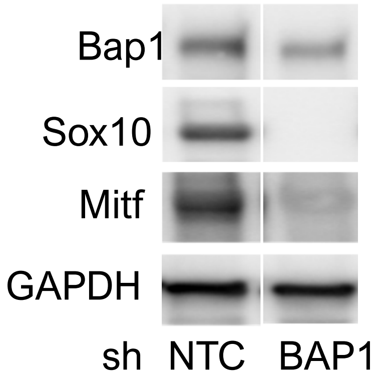

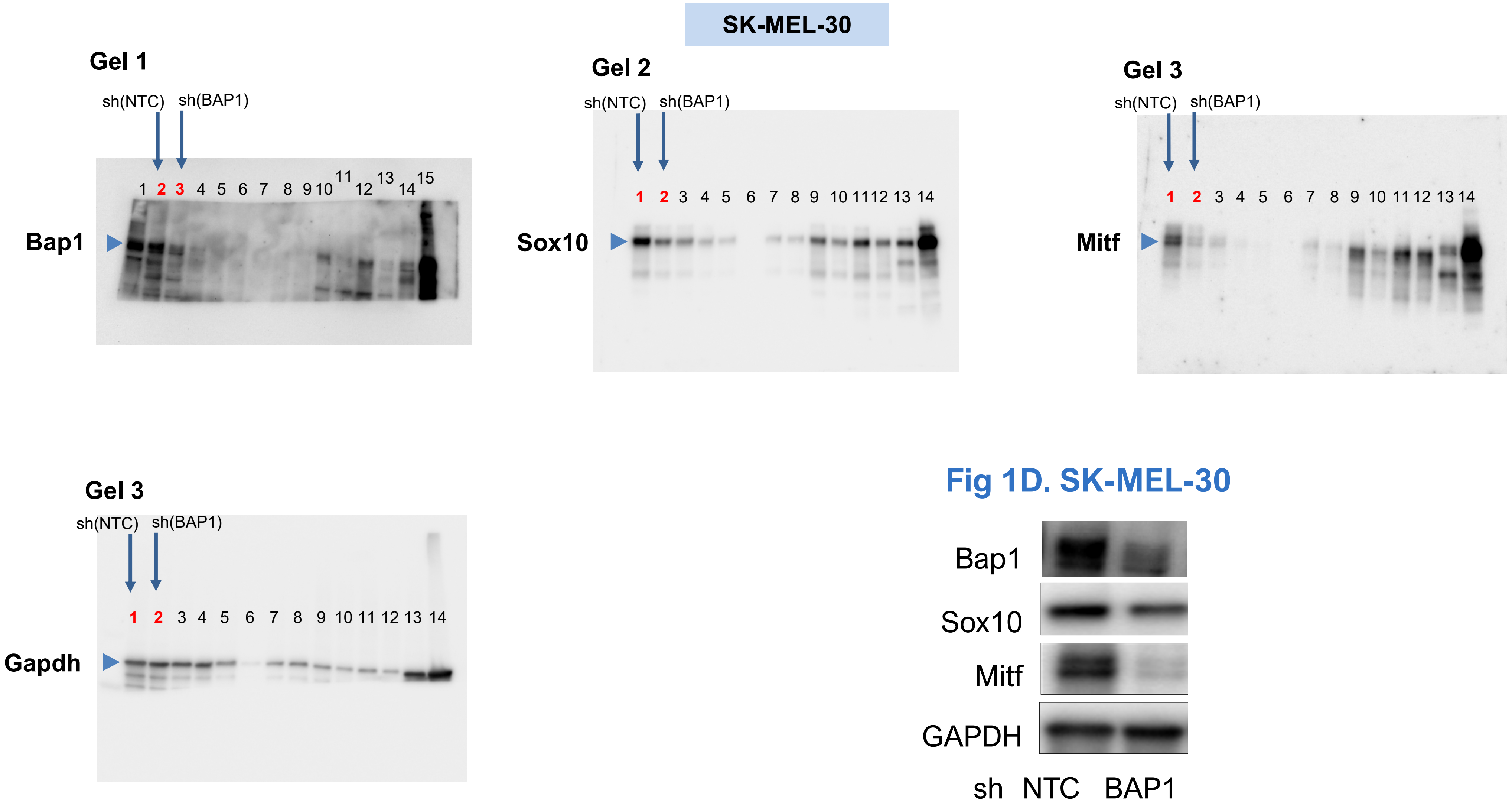
SK-MEL-30
Gel 1
sh(NTC)
sh(BAP1)
1 2 3 4 5 6 7 8 9 10 11 12 13 14 15
Bap1
Gel 2
sh(NTC)
sh(BAP1)
1 2 3 4 5 6 7 8 9 10 11 12 13 14
Sox10
Gel 3
sh(NTC)
sh(BAP1)
1 2 3 4 5 6 7 8 9 10 11 12 13 14
Mitf
Gel 3
sh(NTC)
sh(BAP1)
1 2 3 4 5 6 7 8 9 10 11 12 13 14
Gapdh
Fig 1D. SK-MEL-30
Bap1
Sox10
Mitf
GAPDH
sh NTC BAP1

IGR-37

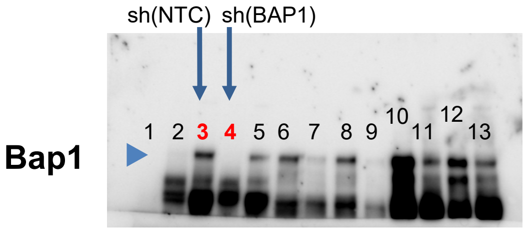

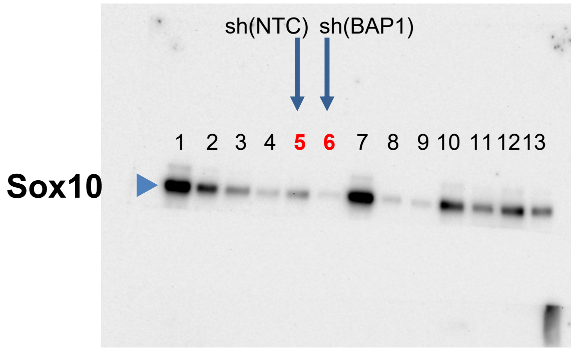

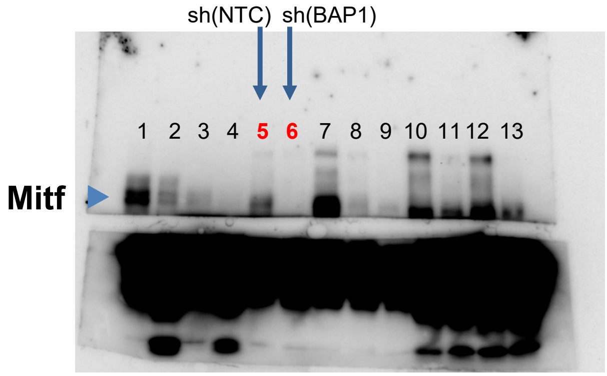

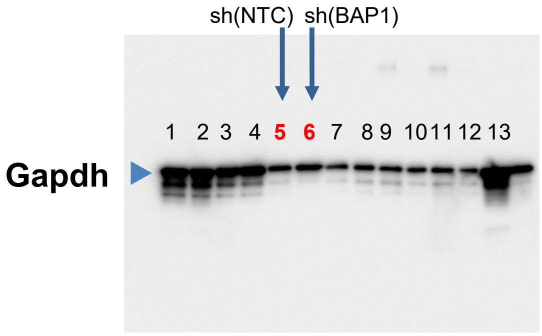

Fig 1D. IGR-37

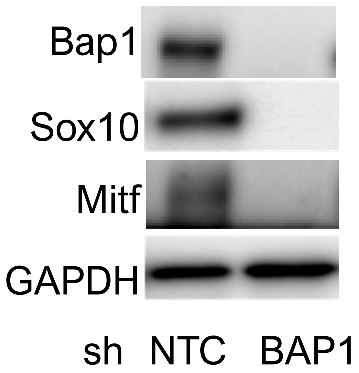

Supplement: Unedited blot and gel images [file jci-136-195809-s337.pdf]
